# Effects of Bovine *Pichia kudriavzevii* T7, *Candida glabrata* B14, and *Lactobacillus plantarum* Y9 on Milk Production, Quality and Digestive Tract Microbiome in Dairy Cows

**DOI:** 10.3390/microorganisms10050842

**Published:** 2022-04-20

**Authors:** Yali Ji, Xin Dong, Zhimin Liu, Weijun Wang, Hai Yan, Xiaolu Liu

**Affiliations:** School of Chemistry and Biological Engineering, University of Science and Technology Beijing, Beijing 100083, China; jiyali0414@163.com (Y.J.); dxzyfx@163.com (X.D.); liuzhimin_1@126.com (Z.L.); 17888803570@163.com (W.W.)

**Keywords:** microbial community, milk production, yeast, lactic acid bacteria, dairy cows

## Abstract

Microbial administration has been used successfully to improve host health. However, the positive effects of endogenous microbials are still underexplored. This study investigated the effects of bovine Lactic acid bacteria and yeast on the milk production, quality and digestive tract microbiome of dairy cows. *Lactobacillus plantarum* Y9, *Pichia kudriavzevii* T7 and *Candida glabrata B14* isolated from high-yielding dairy cows were selected to feed low-yielding Holstein cows. *Pichia kudriavzevii* T7 could significantly increase milk yield, meanwhile, *Pichia kudriavzevii* T7 and *Candida glabrata* B14 could obviously reduce the number of somatic cell counts (SCC). However, slight differences were found in milk fat, protein, lactose and SNF (solids not fat) percentage. High throughput sequencing showed that the dominant bacteria were *Prevotella* and *Ruminococcaceae* in rumen and feces, respectively, and the dominant fungi were *Penicillium*, *Aspergillus* and *Trichoderma* in both samples, before and after feeding the microbial addition. Nonetheless, microbial addition changed the abundance and structure of the microbiome in the digestive tract. Our data showed bovine yeast and LAB were beneficial for improving performance and regulating the microbial structure of dairy cows. This study was expected to enrich the knowledge of the digestive tract microbiome in dairy cows and provide a feasible strategy for the further utilization of bovine microorganisms.

## 1. Introduction

The application of microbial addition is an effective strategy to improve animal performance, productivity, and health in the livestock industry [1]. The additives could inhibit the growth of harmful microorganisms and regulate the enzyme activities related to the metabolism of toxic substances [2]. Furthermore, they could also enhance the resistance to diseases (mastitis and diarrhea, etc.) by improving intestinal barrier function and regulating gut microbiota [3,4,5]. For example, yeast had been effectively utilized for cattle with positive results of dry matter intake (DMI) and milk yield [6]. Additionally, the feeding of mixed *Lactobacillus* administration was also beneficial to the host. They could significantly increase the levels of milk immunoglobulin G (IgG), lactoferrin and lysozyme, decrease somatic cell counts (SCC), and enhance rumen fermentative bacteria and beneficial bacteria, which have beneficial effects in improving the milk yield and quality [7]. However, the specificity of microbial additives and host rejection might weaken the positive effects or even cause a negative result [8].

According to previous studies, when the applied environments are similar to the original, microbes could be more secure and resist population interference effectively. The lactic acid bacteria (LAB), isolated from sheep, had prominent advantages over the other strains in terms of intestinal tolerance and antibacterial properties against pathogens. It was believed to be the best potential candidate [9,10]. As a dominant member of the gut microbial community, diverse LAB is present in the digestive tract of ruminants, such as *Lactobacillus*, *Streptococcus,* and *Enterococcus* [11,12]. The bacteriocins and hydrogen peroxide produced by LAB could inhibit the growth of the *E. coli* and other pathogens in the rumen [13]. Another mechanism of LAB was to enhance the mucosal and systemic immune response for improving body health [11]. *Lactobacillus plantarum* isolated from horse feces showed significant antibacterial activity against pathogenic bacteria, which was expected to be used in the equine industries [14]. Furthermore, the yeast in the rumen of cattle showed a strong viability for promoting rumen function and favorable features to improve volatile fatty acids production [15]. Yeast culture is a common microbial additive practiced in animal husbandry. The inclusion of yeast can contribute to improving animal health, nutrition, and productivity by increasing fiber digestibility and regulating rumen pH [6]. The benefits of yeast also include stimulating the immune system in the intestine [16]. Previous experiments showed that various yeast strains were discovered from the bovine rumen, such as *Pichia kudriavzevii*, *Candida rugosa*, *C. pararugosa* and *C. ethanolica* [15]. Additionally, *Pichia kudriavzevii* isolated from cattle rumen showed great potentiality in terms of the production of biomass and cellulase [16]. Although the beneficial effects of host isolated LAB and yeast have been found, whether the microbes would act on the intestinal microbial structure and organism system effectively is not clear.

The gastrointestinal microbial community of dairy cows is closely associated with host health, nutrient absorption, performance, and feed utilization. Disruption of the microbial balance could lead to digestive tract dysfunction and inflammatory disease, which results in reduced productivity and economic losses [17]. The constitution and dynamic diversity of digestive tract microbes varies with the factors such as health status, host age, dietary pattern and ambient temperature [18]. Zhang et al. reported that the gastrointestinal bacterial composition of Holstein cows changed with increasing age. Compared with older cows, the relative abundance of *Prevotellaceae* was higher in the rumen and feces of young cows [19]. In a study of seasonal changes in the gut microbiome, Nguyen et al. found that the dominant families of the fecal microbiota (*Ruminococcaceae, Bacteroidaceae*, etc.) were not affected by seasons, while the content of *Staphylococcaceae* and *Methylobacteriaceae* were different in the summer and winter [20]. Furthermore, since the feed provided to the rumen and hindgut may differ, the microbiome in the rumen and feces are likely also different [21]. Therefore, studying the digestive tract microbiome of dairy cows can further analyze the positive effects of microbial additives.

In this study, *Lactobacillus plantarum* Y9 (LAB), *Pichia kudriavzevii* T7 and *Candida glabrata* B14 (yeast), isolated from the rumen of high-yield dairy cows, were used as the microbial addition to feed animals. The objective of this experiment was to explore the effects of bovine yeast and LAB in terms of milk yield and the components of dairy cows. In addition, high-throughput sequencing technology was used to analyze the differences of the microbial communities in rumen and feces after feeding different microbial additives. We hypothesized that bovine yeast and LAB would increase milk production, improve milk quality and modulate the digestive tract microbiome of cows. Finally, this study was expected to enrich the knowledge of the digestive tract microbiome in dairy cows and provide a basis for the application of bovine yeast and LAB.

## 2. Materials and Methods

### 2.1. Animals, Diet and Treatments

All the experimental animals were provided by Gansu Tianchen animal husbandry Co., Ltd., Lanzhou, China. The procedures involving animals were approved and conducted according to the standards of the Institutional Animal Care and Use Committee of the Lanzhou Institute of Husbandry and Pharmaceutical Sciences of Chinese Academy of Agricultural Sciences. Thirty-six mid lactating Chinese Holstein cows (age 3 years old, milk yield about 23 kg) were housed in individual pens with free access to water. Six high-yielding cows (age 3 years old, milk yield about 60 kg) were also raised under the same conditions. All cows were fed the same basal diet as a total mixed ration (TMR), without previous administration of antibiotics in the last three months. The TMR composition is shown in Table 1.

Thirty-six Holstein cows were randomly assigned to four groups: one control (CK) and three treatments (G: Feeding *Candida glabrata* B14, P: Feeding *Pichia kudriavzevii* T7, L: Feeding *Lactobacillus plantarum* Y9). Each group consisted of nine cows. The experimental group was fed with three kinds of live microbial preparation every day, each treated animal received 2 × 10^9^ CFU/g/day (CFU, colony forming units) live microbial mixed with the basal diet. The control group was fed with an ordinary diet and freeze-dried protectant (skim milk) [22]. Three kinds of live strain powder were directly fed into the feeding trough of the experimental cows to ensure that the cows were fully fed. The treatment period lasted for 30 days.

### 2.2. Strains Addition

The rumen fluid of cows with high yield was collected for strain screening. Sequential 10-fold sample dilutions were prepared in triplicate, using 0.75% sterile saline solution (weight/volume, *w*/*v*). Then, 1 mL of the sample dilution was spread over De Man Rogosa Sharpe (MRS) agar medium and incubated for 48 h at 38 °C to isolate LAB [23]. Yeasts were cultured in solidified yeast extract peptone-dextrose medium (YPD) at 28 °C for 72 h [15]. Screening strains were identified by purification and standard morphological analysis. DNA was extracted using the Genomic DNA Isolation Kit and was amplified by PCR, followed by high-throughput sequencing for molecular identification. Finally, one strain of LAB and two strains of yeast were obtained, named *Lactobacillus plantarum* Y9, *Pichia kudriavzevii* T7 and *Candida glabrata* B14 (Figure S1). These living microorganisms were fermented and cultured. Then the live bacterial powder was prepared by a freeze-drying method in our laboratory, and was sealed and stored at 4 °C until use. After the preparation, the active microorganism preparations were added to feed.

### 2.3. Milk Sampling and Analysis

Milk production and quality were analyzed according to the previous study [7]. Cows were milked twice daily under hygienic conditions in the bullpen at 8:00 am and 8:00 pm, by hand milking. The milk yield was recorded every day, and nine cows were selected each time. Milk samples (approximately 50 mL) from two milked of each cow were collected by volume on the first day of the trial (before feed administration), and on day 10, day 20 and day 30. Samples were stored at 4 °C until analysis. The fat, protein, lactose and solids not fat (SNF) were determined by MilkioScan^TM^ FT120 (MilkoScan Type 71210, Foss Electric, Hillerød, Denmark), and the somatic cell counts (SCC) were determined by Fossomatic^TM^ Minor (Fossomatic Type 71210, Foss Electric, Hillerød, Denmark), following the manufacturer’s instructions.

### 2.4. Ruminal Samples and Fecal Samples Collection

Nine cows in each group were selected for collecting ruminal fluid samples. The ruminal fluid samples were extracted at day 30 (post microorganisms administration) about 2 h after feeding with an esophageal probe and were then squeezed through four layers of cheesecloth [24]. Finally, 10–20 mL of sample was obtained. Similarly, fresh fecal samples of nine cows in each group (the same as above) were collected and put into a 50 mL sterile tube. The ruminal fluid samples and fecal samples were stored at −20 °C until analysis.

### 2.5. Genomic DNA Extraction and PCR Amplification

After genomic DNA was extracted, specific primers were synthesized for Miseq high-throughput sequencing. Bacterial sequences used primers 515F (5′-GTGCCAGCMGCCGCGG-3) and 907R (5′-CCGTCAATTCMTTTRAGTT-3′), fungal sequences used primers ITS1-1737F (5′-GGAAGTAAAAGTCGTAACAAGG-3′) and ITS2-2043R (5′-GCTGCGTTCTTCATCGATGC-3′). PCR amplification was performed using TransStart^®^ Fastpfu DNA polymerase (Beijing, China) under the following cycling conditions: 5 min at 94 °C, 25 cycles of 45 s at 95 °C, 30 s at 55 °C, 60 s at 72 °C, and 72 °C for 10 min. The PCR products of the same sample were mixed and detected by 2% agarose gel electrophoresis and were recovered by an AxyPrep DNA Gel Recovery Kit (Axygen, Union City, CA, USA).

### 2.6. Biocomputational and Statistical Analyses

The samples were sequenced using MiSeq (Illumina, San Diego, CA, USA) to obtain Pair-end (PE) reads; PE reads were merged by FLASH and then the quality filtering and optimization were performed by Trimmomatic [25]. The data processing results are shown in Table S1. Operational taxonomic units (OTU) cluster analysis and species classification analysis were performed after distinguishing the samples. OTU were clustered using the software platform Usearch (version 7.1 146, http://drive5.com/uparse/, accessed on 14 March 2021). After extracting the nonrepetitive sequences from the optimized sequences, OTU clustering was performed based on 97% similarity, and chimera were removed during the clustering process to obtain the representative OTU sequences. Then, all valid sequences were mapped to the representative OTU sequences to obtain the OTU cluster analysis results. Venn diagrams were used to display the number of shared OTUs between the control and experimental cows, and were constructed using the jvenn tool [26]. The RDP classifier Bayesian algorithm was used to classify OTU representative sequences, and the community composition of each sample was calculated at phylum and genus levels [27]. The diversity indices (Shannon, Simpson, Chao and Ace indices) were performed using Mothur for evaluating the sequence depth, biodiversity and richness.

Statistical analysis was performed using Origin 2019 and SPSS 23.0. The effects of microbial addition on milk yield and quality were further analyzed by a univariate ANOVA for milk yield, composition and SCC of the control and experimental cows. Differences between means of measurement data were defined as significant at *p* < 0.05.

## 3. Results

### 3.1. Milk Production and Components

**Figure 1:**
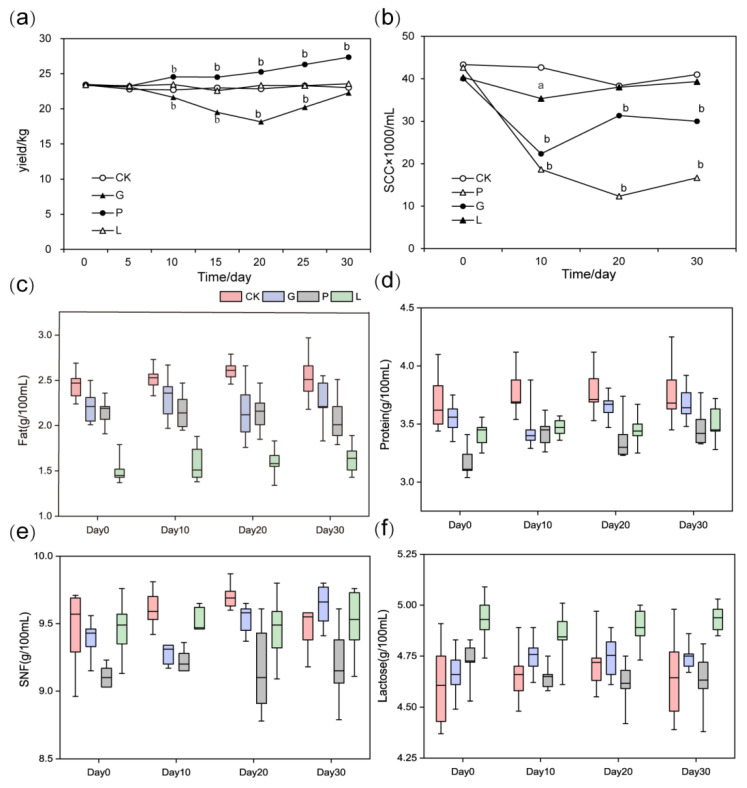
Effects of different microbial treatments on milk production (**a**), SCC (**b**) and milk quality. (**c**) Fat content in milk, (**d**) Protein content in milk, (**e**) SNF content in milk, (**f**) Lactose content in milk. a: Compared with the control group, *p <* 0.05, b: Compared with the control group, *p <* 0.01.

The milk yield of lactation was similar at the start of the experiment; about 23 kg. During the whole experimental period (5d–30d), the milk production of the *Pichia kudriavzevii* T7 group was significantly higher than that of the control group, and it increased by 4.3 kg on the 30th day (14.15% increment, *p* < 0.01). When added to the *Candida glabrata* B14, production of milk decreased significantly on the 10th, 15th, and 20th days in comparison to the control group, with a maximum decrease of 22.17% on the 20th day (*p* < 0.01), and then gradually increased. No significant differences in milk yield were found between *Lactobacillus plantarum* Y9 treated cows and the control cows (Figure 1a). Compared with untreated cows, milk SCC was significantly decreased with the consumption of *Pichia kudriavzevii* T7 (*p* < 0.01). The *Candida glabrata* B14 treatment also showed a considerably lower content of SCC during the trial period (day 10 to day 30, *p* < 0.01). However, no significant difference was observed in SCC after feeding *Lactobacillus plantarum* Y9 compared with the control group (*p* > 0.05, Figure 1b). Note that the proportions of milk protein, fat, lactose and SNF did not differ significantly for all animals at any time points (*p* > 0.05, Figure 1c–f).

### 3.2. Microbial Diversity Analysis

**Figure 2:**
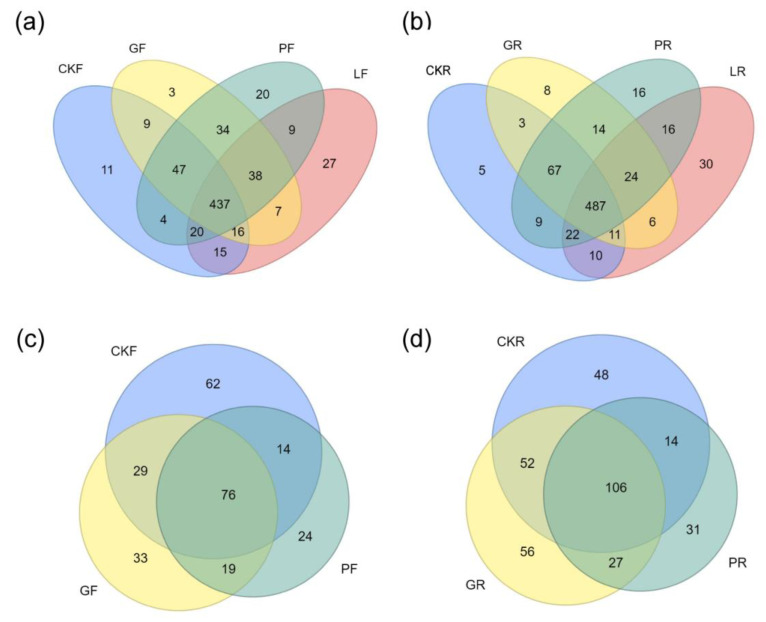
The OTU number of bacteria and fungi in different treatment groups. (**a**) Bacteria OTUs in feces, (**b**) Bacteria OTUs in rumen, (**c**) Fungal OTUs in feces, (**d**) Fungal OTUs in rumen. R: rumen samples, F: feces samples.

The Venn diagram showed that the number of bacteria was higher than that of fungi. Subject cows fed with *Lactobacillus plantarum* Y9 demonstrated the largest number of bacterial OTUs and possessed the most unique bacteria OTUs (Figure 2a,b). Compared to other treatments, the fungal communities in the rumen of *Candida glabrata* B14 intervention identified the largest number of OTUs (Figure 2d).

**Figure 3:**
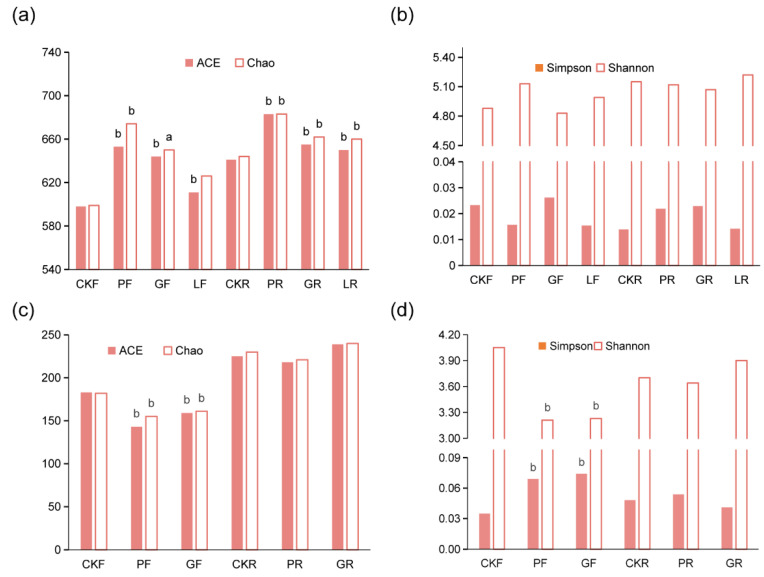
Diversity index of bacterial and fungal microbiome in the rumen and feces of dairy cows under different treatments. (**a**) ACE index and Chao index of bacterial microbiome, (**b**) Shannon index and Simpson index of bacterial microbiome, (**c**) ACE index and Chao index of fungal microbiome, (**d**) Shannon index and Simpson index of fungal microbiome. F: feces samples. R: rumen samples. a: Compared with the control group, *p* < 0.05, b: Compared with the control group, *p* < 0.01.

In this experiment, the diversity indices were used to reflect the abundance, evenness, and diversity of the microbiome in rumen and feces. The Chao and ACE indices of the samples receiving bovine yeast and LAB were significantly higher than those of samples without treatment (Figure 3a). It is notable that the microbial richness in the digestive tract was highest during the application of *Pichia kudriavzevii* T7 (Figure 3a). This result indicated that bovine microorganisms improved the bacterial richness in the digestive tract after 30 days. Additionally, the microbial diversity (Shannon index, Simpson index) of rumen and fecal bacteria was slightly changed in cows with bovine yeast and LAB supplemented in comparison with the corresponding non-treatment animals (Figure 3b). Figure 3 also showed the diversity of fungi in rumen and feces. The levels of ACE and Chao in fecal samples fed *Pichia kudriavzevii* T7 and *Candida glabrata* B14 were lower than those in control animals. No significant difference was observed in ACE and Chao indices of rumen microorganisms between control and yeast intervention cows (Figure 3c). Furthermore, bovine yeast significantly increased the species diversity (Shannon and Simpson indices) of the fecal microbiome as compared with the control, while it did not have a substantial effect on the rumen microbiome (Figure 3d).

### 3.3. Analysis of Bacterial Microbial Compositio

**Figure 4:**
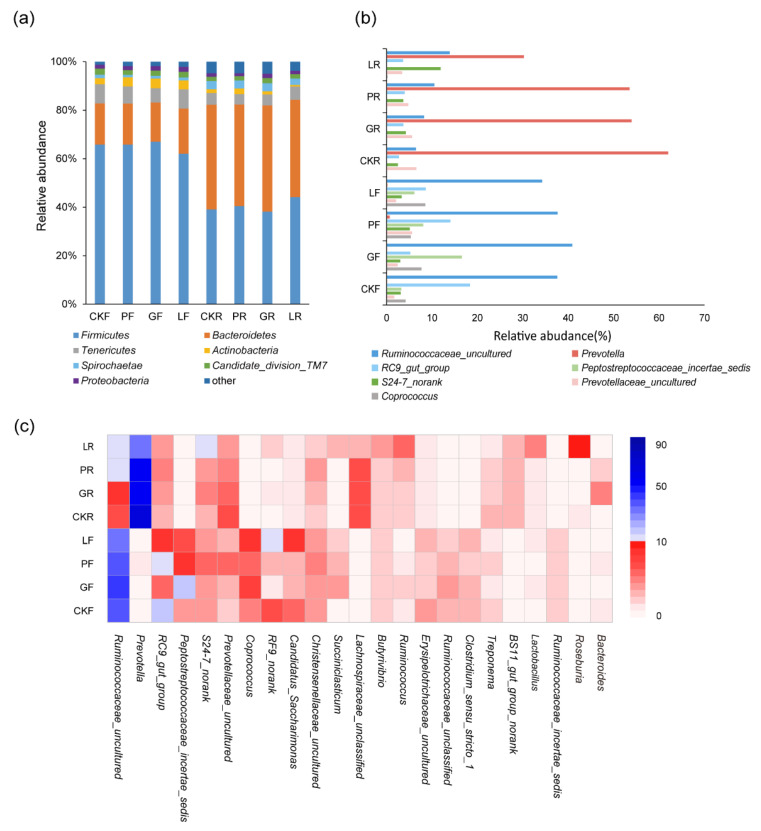
The influence of different treatments on the bacterial community structure in the rumen and feces of dairy cows. (**a**) The bacterial community structure composition at phylum level, (**b**) Relative abundance of dominant genera, (**c**) The top 23 bacterial genera in relative abundance in dairy cow samples. F: feces sample. R: rumen sample.

Figure 4 revealed the bacterial community differences in rumen and fecal samples. At the phylum level, the active bacterial community in the rumen consisted of 18 phyla, of which *Firmicutes* and *Bacteroidetes* were the two predominant phyla, with a relative abundance of about 80% in the rumen and feces. After 30 days of bovine microbial treatment, no statistically significant difference was observed in the relative proportions between treated and control groups in the rumen and fecal samples (Figure 4a). At the genus level, the composition of bacteria in rumen was predominated by the genus *prevotella*, *Ruminococcaceae_uncultured* and *Succiniclasticum* (Figure 4b). Notably, the genus *prevotella* were significantly reduced to 30% after feeding *Lactobacillus plantarum* Y9, while representing about 60% of all sequences in the other treatments. An opposite change trend was observed in the *Ruminococcaceae_uncultured* genus. Further, *Butyrivibrio* and *Ruminococcus* accounted for 1–5% of the populations. About 1% common rumen bacteria such as *Lachnospiraceae, Lactobacillus* and *Treponema* was also observed. Interestingly, the content of *Ruminococcaceae_uncultured* and *Peptostreptococcaceae* in fecal groups improved significantly, accounting for 37% and 8% respectively. The *Prevotella* genus almost disappeared in the fecal samples with a relative richness lower than 1%. In addition, the relative abundance of *Peptostreptococcaceae_incertae_sedis* in *Candida glabrata* B14 supplemented cows was higher than those animals on a normal diet. A similar growing trend was observed in *Coprococcus* of *Candida glabrata* B14 and *Lactobacillus plantarum* Y9 administrated cows. Conclusively, bovine LAB and yeast changed the relative abundance of prevalent bacteria in the rumen and feces, but did not change their dominant position.

### 3.4. Analysis of Fungi Microbial Composition

**Figure 5:**
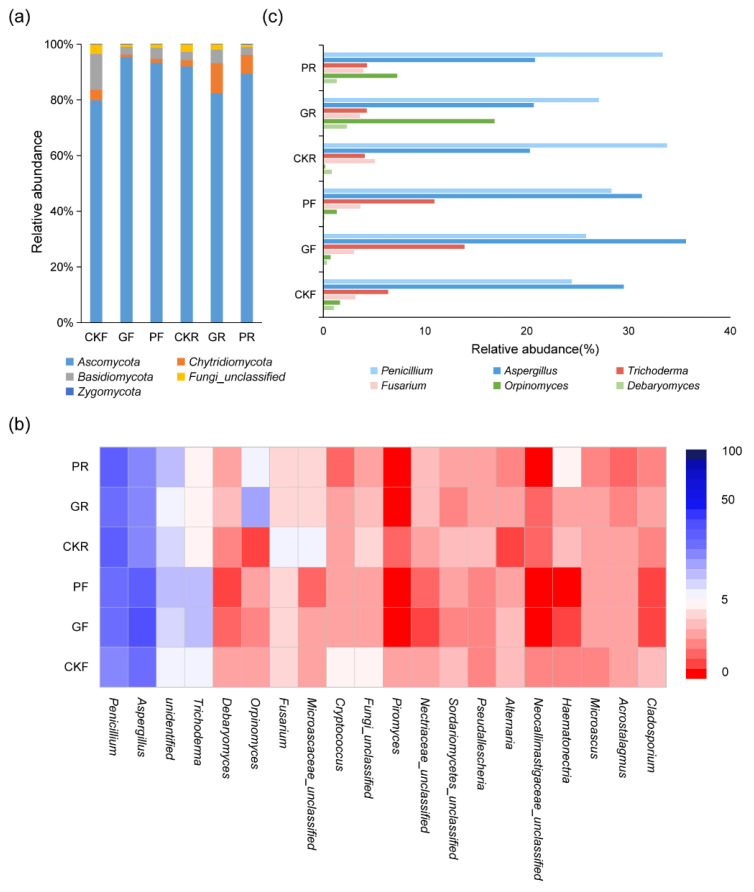
The influence of different treatments on the fungal community structure in the rumen and feces of dairy cows. (**a**) The fungal community structure composition at phylum level, (**b**) The top 20 fungal genera in relative abundance in dairy cow samples, (**c**) Relative abundance of dominant genera. F: feces sample. R: rumen sample.

Five fungal phyla were identified in the experimental samples. *Ascomycota* was the dominant phylum in the rumen and feces with the highest relative abundance. *Basidiomycota* and *Chytridiomycota* were also found be present in the samples (Figure 5a). At the genus level, the main fungal microbial community of rumen and feces were similar, mainly *Penicillium*, *Aspergillus*, *Trichoderma*, *Debaryomyces*, *Orpinomyces,* and *Fusarium* (Figure 5c). Three kinds of mold (*Penicillium*, *Aspergillus*, *Trichoderma*) were the dominant genus in the experimental animals, accounting for 52–75% of the total microbial population. There was no significant difference in the proportion of *Fusarium* among each subject cow (3–5%). Compared with the other samples, an increase in the relative abundance of *Orpinomyces* in rumen was commonly observed in cows that received 30 days of *Candida glabrata* B14 and *Pichia kudriavzevii* T7 intervention (16.8%, 7.3%). Experimental cows receiving *Candida glabrata* B14 were characterized by higher relative numbers of *Debaryomyces* in rumen, accounting for 2%. Conversely, the content of *Debaryomyces* was less than 1% in the other samples. In brief, bovine yeast caused an increase in the relative abundance of dominant fungi in the digestive tract of dairy cows.

## 4. Discussion

Several microbial interventions, including yeast and *Lactobacillus* addition, have been designed to promote host health and boost the production performance of dairy cows [28,29]. Nevertheless, most of the commonly used strains at present are exogenous microorganisms. Hence, the positive impact of microorganisms isolated from the host on itself was not sufficiently investigated. In this study, three strains selected from the rumen of high-yield dairy cows were used as the research objects. The aims of our study were to explore the effects of bovine microorganisms on milk production, quality, and microbial communities in the digestive tract of Holstein cows.

After 30 days of the feeding experiment, *Pichia kudriavzevii* T7 generated a beneficial effect of on milk production, which was in agreement with the previous studies [30,31]. Hence, the yeast might be related to the improvement in lactation performance of ruminants. Silage could be fermented to produce large amounts of lactic acid during the formation [32]. This experimental feed contained a large amount of silage, which means the lactic acid level in the control and experimental groups will not change obviously due to the ingestion of *Lactobacillus plantarum* Y9. This may be the reason why the addition of *Lactobacillus plantarum* Y9 in the diet has no significant correlation with milk production. However, several studies had indicated that, in the dairy industry, *Lactobacillus plantarum* Y9 played an important role in improving the fermentation characteristics of milk [31,33]. In the *Candida glabrata* B14 group, milk yield was lower than that of the control group in the early stage, and then increased considerably (Figure 1a). This phenomenon may be due to the ingestion of *Candida glabrata* B14 causing an imbalance of gastrointestinal microecology in dairy cows. Nevertheless, it might be noticed that the imbalance could gradually recover after a period of adaptation. Hence, whether *Candida glabrata* B14 will increase milk production after adaptation remains to be further studied.

In this trial, both yeasts could significantly suppress the SCC, while *Lactobacillus plantarum* Y9 had no obvious effect (Figure 1b). SCC is an assessment index of mastitis reflecting the cattle health and milk quality. Cows with high SCC are more likely to possess a high bacterial load and serve as a reservoir of pathogens in healthy animals [34]. Mastitis is the most prevalent disease in cattle breeding, which has a serious impact on animal welfare and economic benefits [35]. The significant reduction of SCC value indicated that bovine yeast might effectively ameliorate breast inflammation of dairy cows through inhibiting pathogenic bacteria, and subsequently produced more economic benefits. Besides, the major milk composition, including protein, fat, lactose and SNF, remained at a similar value before and after microbial treatment (Figure 1). Some previously reported studies have reached inconsistent conclusions. Ma and colleagues found that milk fat, protein, and lactose percentage tend to be higher in ruminants supplemented with probiotics [36,37,38]. Another study found no obvious changes in the milk components after the inclusion of yeast [39]. Possibly, different elements contributed to this discrepancy, since the trials were implemented under different experimental conditions. Additionally, the physiological stage of experimental animals (age, lactation period, milk yield, etc.) and ingredients of the diet were also different. Another possible reason was the difference in the properties, types and source of strains relating to the experiments. Although our results indicated that microbial addition had no substantial impact on milk quality, an increase in milk production would still reduce production costs and increase economic benefits.

The rumen microbiome is of great importance for rumen fermentation and milk production [40]. The OTU cluster displayed that the bacterial population possessed a high classification stability (75%, Figure 2). Rumen and fecal bacterial richness were significantly increased after feeding microbial addition, while the dietary addition of microorganisms did not cause a significant change in the bacterial diversity (Figure 3a,b). In contrast, bovine yeast apparently reduced the richness and diversity of fungi in the feces, but had little effect on the rumen microbiome (Figure 3c,d). Such results suggested that bovine yeast and LAB could promote the better growth and reproduction of different bacteria. This might be due to microbial additives changing the digestive tract microbial environment of dairy cows.

Furthermore, we investigated the effect of the bovine yeast and LAB intake on the microbiome in the digestive tract of dairy cows. In the present study, *Firmicutes* was the most dominant phylum in the ruminants, as in other previous results [36]. *Bacteroidetes* was also often observed as another prevalent phylum in dairy cows [41]. *Ascomycota* and *Basidiomycota* were the main fungal phyla, which represented the main fungal decomposers in livestock manure [42]. The relative abundance of each phylum remained stable, indicating that bovine microorganisms had little effect on the community structure at the phylum level. At the genus level, *Prevotella* is the most prevalent genus in rumen, which is in agreement with the published study [43,44]. *Prevotella* is extensively present in the intestines of many mammals, such as human, monkey, and mice [45,46,47]. As one of the important members among the rumen microbiome, *Prevotella* spp. make a considerable contribution to the carbohydrates and nitrogen utilization of ruminants [47,48]. Previous research also indicated favorable influences of some *Prevotella* strains on the improvement of immunity [49]. Therefore, *Prevotella* is conducive to the health and growth of dairy cows, which in turn contributes to the improvement of milk production and quality. The core genera also include *Ruminococcaceae_uncultured* and *Succiniclasticum.* The *Ruminococcaceae* has been well described as responsible for degrading many polysaccharides and fibers [50]. Bacteria of the *Succiniclasticum* genus are important converters of succinate to propionate [44]. Ruminants use propionate to produce glucose, so abundant *Succiniclasticum* improve the animal diet utilization [51].

The *Lactobacillus plantarum* Y9 addition significantly increased the relative abundance of *Lachnospiraceae_incertae_sedis* and *Bacteroides* in the rumen microbiota of dairy cows (Figure 4c). *Bacteroides* is one of the ordinary native intestinal bacteria members, and the presence of more *Bacteroides* in the rumen could help animals metabolize oligofructose in plant-derived feed [52]. *Lacetospirillaceae* showed a considerable ability to degrade indigestible fibers, and could hydrolyze starch and other polysaccharides for producing short-chain fatty acids [53,54]. Short-chain fatty acids are involved in maintaining a healthy gut microbiota and enhancing host antibody production [55]. The increase in the abundance of these strains was beneficial to the utilization of feed and the improvement of cattle physiological conditions. Additionally, bovine yeast and LAB ingestion increased *Butyrivibrio* and *Ruminococcus* content in the rumen of dairy cows during the trial (Figure 4c). As well-known rumen cellulolytic bacteria, they play a vital role in the degradation and utilization of plant fibers. Members of the genus *Butyrivibrio* also have capabilities to decompose polysaccharides and ferment degradation products, which are used by ruminants to grow and produce milk [56,57]. These results revealed that dietary addition of bovine yeast and LAB may have a positive effect on the balance of the rumen microbiome at the genus level, and then contribute to the digestion and health of dairy cows.

As for feces, the abundance of microbial components has obviously changed. Some strains of *Peptostreptococcus* produce indoleacrylic acid to strengthen the gut barrier and inhibit animal inflammatory diseases [58]. *Coprococcus* bacteria are participants in multiple metabolic pathways in the ruminant rumen. They can improve feed efficiency and reduce CH_4_ emissions [59]. The high abundance of these bacteria indicated that microbial addition is beneficial for improving gut health and increasing economic benefits. After a period of feeding with *Lactobacillus plantarum* Y9, the fecal microbiome had a more balanced bacterial structure compared with other processed samples. *Lactobacillus plantarum* Y9 is a kind of lactic acid bacteria, which can efficiently participate in carbohydrate metabolism and fermentation [60,61]. Another capability of this strain is to producer diverse bacteriocins for inhibiting the growth of food-borne pathogens and promoting the proliferation of gut probiotics [62]. Moreover, *Lactobacillus plantarum* Y9 reduced inflammation and relieved diarrhea through regulating the intestinal microbiota [1,5]. These characteristics of *Lactobacillus plantarum* Y9 may be one reason for changing the intestinal microbial community and regulating the microecological balance. This is consistent with previous studies finding that *Lactobacillus plantarum* Y9 contributed to regulating the structure of intestinal microbes and promoting metabolism and body health [5,60]. However, the bacterial community structure after feeding yeast was closer to that of the control group. This is inconsistent with previous studies finding that yeast altered the composition of gut microbiota [63,64]. What caused this difference remains to be further studied.

In terms of fungus, the number of *Orpinomyces* and *Debaryomyces* was increased within the rumen of cows receiving the *Candida glabrata* B14 and *Pichia kudriavzevii* T7 treatment. *Debaryomyces,* a kind of yeast, was able to modulate gut microbiota to treat diarrhea and stimulate the expression of immune factors [65,66]. Our results illustrated that the addition of bovine yeast was conducive to the health and nutrient utilization of dairy cows. It might be that yeast shifted the rumen environment to promote better proliferation of *Debaryomyces*. Anaerobic fungi were common microorganisms in the gastrointestinal tract of ruminants and monogastric herbivores, such as *Piromyces* and *Orpinomyces* [67]. The sequencing results of camel rumen fungi revealed that anaerobic fungi could produce corresponding enzymes to degrade fiber and xylan [68]. Other evidence indicated that anaerobic rumen fungi were associated with the improvement of nutrient digestibility, rumen fermentation, and milk production [69]. *Orpinomyces* plays a crucial role in aspects of degrading lignin-rich plant biomass and the improvement of the production performance of animals, representing a desirable gut microbe for providing bioenergy and promotion of animal growth [70,71]. These observations suggested that cows fed with *Candida glabrata* B14 and *Pichia kudriavzevii* T7 probably had more potential to utilize stubborn materials and promote growth.

## 5. Conclusions

Overall, this study demonstrated that feeding *Pichia kudriavzevii* T7 was able to significantly enhance milk production, meanwhile, *Pichia kudriavzevii* T7 and *Candida glabrata* B14 could reduce the SCC. However, bovine yeast and LAB had no effect on the content of protein, fat, lactose and SNF in milk. Furthermore, bovine LAB and yeast did not induce significant changes in the digestive tract microbiome, but changed the abundance of microorganisms. Note that *Lactobacillus plantarum* Y9 was beneficial to the balance of the bacterial community in the digestive tract of dairy cows. Our research showed that the application of host-derived microorganisms to the original environment can improve the physiological performance of the host. This provides a strategy for effectively exploiting the benefits of bovine yeast and LAB.

## Figures and Tables

**Table 1:** Ingredient and chemical composition (mean) of the TMR.

Item	Content (%)	Item	Content (%)
Corn silage	55.0	Soybean meal	2.2
Corn kernels	15.0	Peanut meal	2.0
Alfalfa hay	10.0	Stone powder	0.7
Beet granules	4.5	NaHCO_3_	0.5
Wheat bran	3.2	NaCl	0.3
Oat grass	3.0	Ca(HCO_3_)_2_	0.3
Cottonseed meal	3.0	Special premix	0.3

## Data Availability

Not applicable.

## Supplementary Materials

The following supporting information can be downloaded at: https://www.mdpi.com/article/10.3390/microorganisms10050842/s1, Figure S1: Phylogenetic tree of strains isolated from the rumen of high-yielding dairy cows. (a) Lactobacillus plantarum Y9, (b) Pichia kudriavzevii T7, (c) Candida glabrata B14. Table S1: High throughput sequencing of bacteria and fungus.
