# Supplementary material for: Effects of Bovine Pichia kudriavzevii T7, Candida glabrata B14, and Lactobacillus plantarum Y9 on Milk Production, Quality and Digestive Tract Microbiome in Dairy Cows
Source: Microorganisms. 2022 Apr 20;10(5):842. doi: 10.3390/microorganisms10050842 (PMC9146454; doi:10.3390/microorganisms10050842)
Supplement: Supplementary file 1 [file microorganisms-10-00842-s001.zip › Figure S1.pdf]

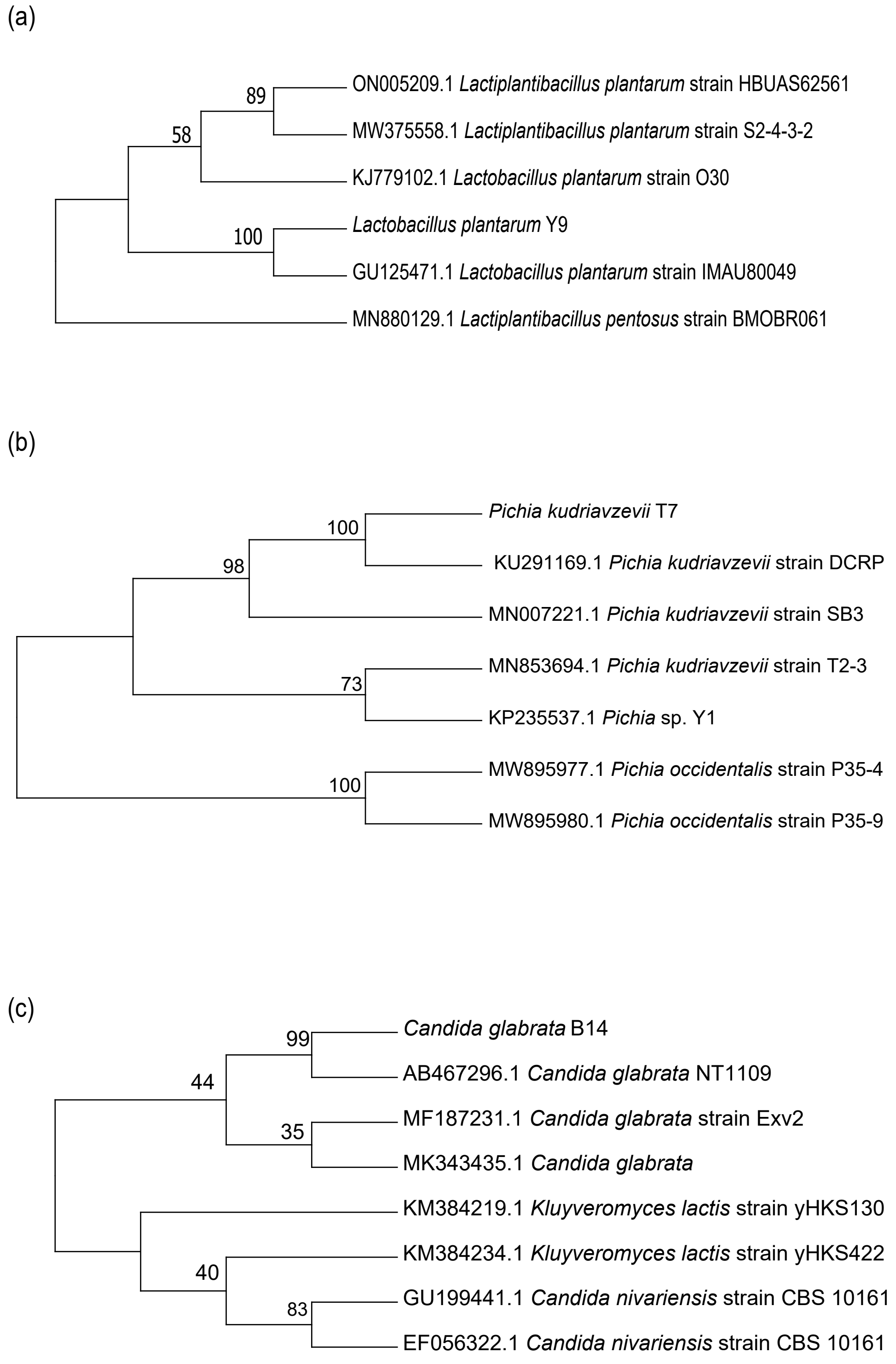

Figure S1. Phylogenetic tree of strains isolated from the rumen of high-yielding dairy cows. (a): *Lactobacillus plantarum* Y9, (b): *Pichia kudriavzevii* T7, (c): *Candida glabrata* B14.
