# Supplementary material for: Effects of Bovine Pichia kudriavzevii T7, Candida glabrata B14, and Lactobacillus plantarum Y9 on Milk Production, Quality and Digestive Tract Microbiome in Dairy Cows
Source: Microorganisms. 2022 Apr 20;10(5):842. doi: 10.3390/microorganisms10050842 (PMC9146454; doi:10.3390/microorganisms10050842)
Supplement: Supplementary file 1 [file microorganisms-10-00842-s001.zip › Table S1.pdf]

Supplementary files

Supplementary Table S1. High throughput sequencing of bacteria and fungus

group	Bacteria			fungus		
	Sequences	Bases(bp)	Average Length(bp)	Sequences	Bases(bp)	Average Length(bp)
CKR	37440	16568581	442.54	31975	8551376	267.44
CKF	36637	15914646	434.39	35561	9757022	274.37
PR	37892	16704420	440.84	40374	10982508	272.02
PF	42063	18236273	433.55	30695	8424203	274.45
GR	33413	14762029	441.8	40374	10982508	272.02
GF	43400	18650045	429.72	30695	8424203	274.45
LR	38147	16667038	436.92	-	-	-
LF	41686	18076473	433.63	-	-	-

Note: R: Rumen samples of cows, F: Feces samples of cows.
